# Cyclodextrin-Based Nanostructure Efficiently Delivers siRNA to Glioblastoma Cells Preferentially via Macropinocytosis

**DOI:** 10.3390/ijms21239306

**Published:** 2020-12-06

**Authors:** Darío Manzanares, María Dolores Pérez-Carrión, José Luis Jiménez Blanco, Carmen Ortiz Mellet, José Manuel García Fernández, Valentín Ceña

**Affiliations:** 1 Unidad Asociada Neurodeath, Universidad de Castilla-La Mancha, 02006 Albacete, Spain; dariomanzafarmacia@gmail.com (D.M.); mdolores.perez.carrion@gmail.com (M.D.P.-C.); 2 CIBERNED, Instituto de Salud Carlos III, 28029 Madrid, Spain; 3 Departamento de Química Orgánica, Facultad de Química, Universidad de Sevilla, 41012 Sevilla, Spain; jljb@us.es (J.L.J.B.); mellet@us.es (C.O.M.); 4 Instituto de Investigaciones Químicas (IIQ), Consejo Superior de Investigaciones Científicas-Universidad de Sevilla, 41092 Sevilla, Spain, jogarcia@iiq.csic.es

**Keywords:** siRNA, β-cyclodextrin, endocytosis, transfection efficiency, micropinocytosis

## Abstract

Small interfering ribonucleic acid (siRNA) has the potential to revolutionize therapeutics since it can knockdown very efficiently the target protein. It is starting to be widely used to interfere with cell infection by HIV. However, naked siRNAs are unable to get into the cell, requiring the use of carriers to protect them from degradation and transporting them across the cell membrane. There is no information about which is the most efficient endocytosis route for high siRNA transfection efficiency. One of the most promising carriers to efficiently deliver siRNA are cyclodextrin derivatives. We have used nanocomplexes composed of siRNA and a β-cyclodextrin derivative, AMC6, with a very high transfection efficiency to selectively knockdown clathrin heavy chain, caveolin 1, and p21 Activated Kinase 1 to specifically block clathrin-mediated, caveolin-mediated and macropinocytosis endocytic pathways. The main objective was to identify whether there is a preferential endocytic pathway associated with high siRNA transfection efficiency. We have found that macropinocytosis is the preferential entry pathway for the nanoparticle and its associated siRNA cargo. However, blockade of macropinocytosis does not affect AMC6-mediated transfection efficiency, suggesting that macropinocytosis blockade can be functionally compensated by an increase in clathrin- and caveolin-mediated endocytosis.

## 1. Introduction

RNAi represents an effective mechanism that operates in most eukaryotic cells [1] regulating the activity of miRNAs and so being involved in the regulation of cellular metabolism, replication or malignant transformation [2]. RNAi has a high potential to be useful in therapeutics since it is able to knockdown proteins involved in the pathogenesis of different diseases [3], including HIV [4], by targeting cellular mRNA [5]. Clinical trials have already shown RNAi effectivity in some diseases such as hereditary transthyretin amyloidosis [6] and acute intermittent porphyria [7]. In addition, RNAi-mediated knockdown of proteins involved in cancer cell survival has been proposed to potentiate antitumoral actions of drugs, suggesting a new potential therapeutic approach for cancer treatment [8].

Several exogenous activators of the RNAi system can knockdown specific sequences involved in cellular signaling, small interfering RNAs (siRNAs) being the most widely used [9]. siRNAs are double-strand RNAs each strand formed by about 21 nucleotides that degrade homologous mRNAs [10]. siRNAs can knockdown very efficiently the proteins encoded by the target mRNAs, but naked siRNAs are unable to get into the cell, requiring the use of carriers capable of forming nanostructures (nanoparticles; NPs), protecting the siRNA molecules from degradation and transporting them across the cell membrane [11].

siRNA-loaded NPs can gain access to the cell interior through simple diffusion or translocation, an energy-independent process that relies on the NP concentration gradient, but also on other factors such as their size, shape, surface charge or lipophilicity [12]. However, the most common mechanism used by NPs to enter the cells is endocytosis [13]. Several endocytosis mechanisms have been described, but the major pathways for endocytic uptake followed by NPs and their cargo to enter the cell are macropinocytosis, clathrin-mediated endocytosis (CME), and caveolin-mediated endocytosis (CVME) [14].

Detailed knowledge of the specific uptake route followed by a given NP is very relevant, since it determines the NP fate as well as the fate of the corresponding cargo, conditioning its possible use as drug delivery system/pharmaceutics. This is even more relevant in the case of siRNA therapeutics since one key step for their efficiency is endosomal escape [15]. In this sense, CVME is generally advantageous since it seems to avoid the endo-lysosomal system in some cell types [16]. Nevertheless, other authors report that macropinosomes are more likely to release their content without lysosomal degradation and thus are preferable for siRNA delivery [17].

For a long time, the study of the different endocytosis pathways has been carried out using different chemical inhibitors to block several endocytic pathways [18,19,20]. However, despite their wide utilization, these compounds are not specific and simultaneously affect several routes [21], which does not allow a precise dissection of the contribution of a given endocytic pathway to siRNA transfection efficiency. A more specific approach would involve knocking down, using specific siRNAs, certain proteins that play essential roles in the different endocytic uptake routes used by NPs. Thus, we have selectively knocked down clathrin heavy chain (CLTC), an integral component of the clathrin triskelion that forms the clathrin coat in CME [22] to block the clathrin-dependent pathway [23]; caveolin 1 (CAV1) to block the caveolin-dependent pathway [23]; and p21 (Rac1) Activated Kinase 1 (PAK1) to block the macropinocytosis pathway, because its knockdown interrupts the route of activation of macropinocytosis after activation of both Rac1 and Cdc42, which through PAK1 induce the polymerization of actin [24].

**Figure 1:**
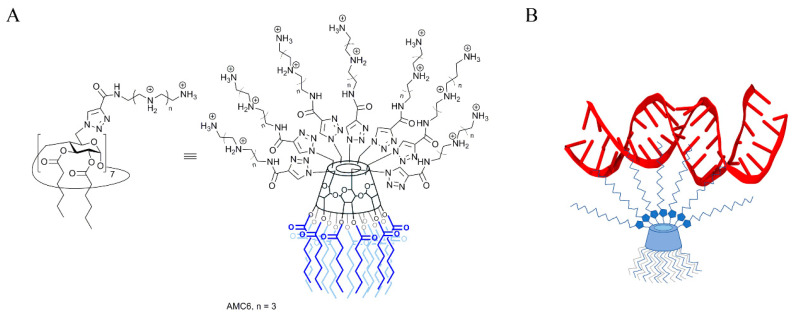
(**A**) Chemical structure of the polycationic amphiphilic β-cyclodextrin derivative AMC6; (**B**) Scheme showing siRNA binding to an AMC6 molecule.

As mentioned above, NPs are required to both protect siRNA from degradation and to deliver it to the cells [11]. To achieve this, we have chosen as carrier a β-cyclodextrin (βCD)-based molecular multivalent amphiphile [25] termed AMC6 (Figure 1). AMC6 is a member of the polycationic amphiphilic cyclodextrin nonviral vector family [26], characterized by a Janus-type architecture with segregated polycationic and lipophilic domains [27]. It consists of a basket-shaped βCD core with 14 hexanoyl chains at the secondary face and seven tetraethyleneimine branches at the opposite primary rim. These branches at the seven primary positions contain a triazol linker and four protonable nitrogens each, which give to the molecule a total of 28 cationizable centers [28]. AMC6 forms self-assembled nanoparticles in the presence of siRNA with an estimated size determined by Dynamic Light Scattering of about 110 nm. AMC6 nanoparticles have previously shown no toxicity and a high transfection efficiency in different tumoral cell lines including human U87 and rat C6 glioblastoma [29].

The results presented here show that AMC6 is an excellent siRNA transfection vector that is able to deliver siRNA to decrease target protein levels to about 15 to 25% of control values, a transfection efficiency than is better than that observed in the case of cationic lipid nanoparticles or calcium phosphate nanoparticles, allowing lack-of-function studies. AMC6-mediated siRNA delivery can knockdown key proteins (CLTC, CAV1, and PAK1) of the major endocytic pathways (CME, CVME and macropinocytosis) leading to a functional blockade of them as seen by the marked decrease in the uptake of molecules such as transferrin and 70 K dextran that are specifically taken up by those pathways. This has allowed the dissection of the contribution of each endocytic pathway to AMC6-mediated siRNA delivery to glioblastoma cells. We have found that the major endocytic pathway in T98G glioblastoma cells is macropinocytosis, although the three major pathways contribute to AMC6 efficient siRNA transfection functionally compensating the blockade of one of them.

## 2. Results

### 2.1. Time-Course of siRNA Uptake and Cell Toxicity

**Figure 2:**
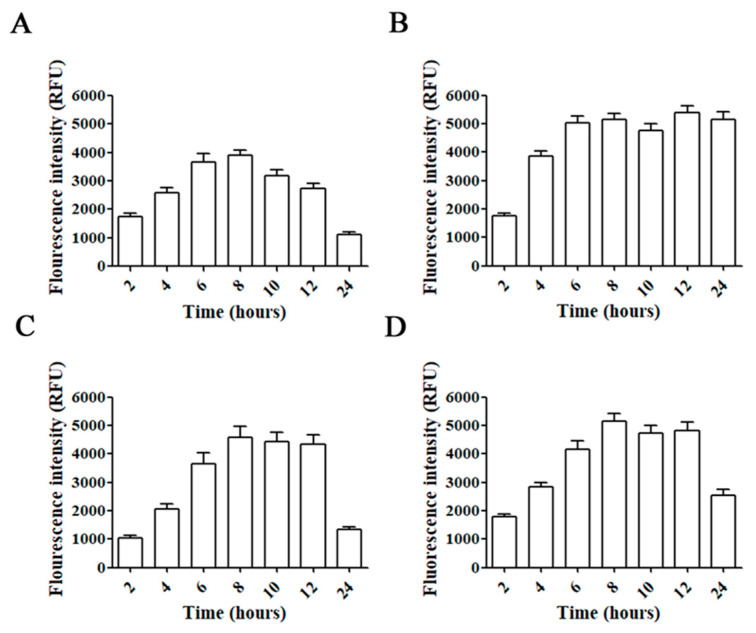
Time-courses for uptake of AMC6/Alexa Fluor 488-labeled siRNA complexes. C6 (**A**), U87 (**B**), GL261 (**C**) and T98G (**D**) cell lines were treated with AMC6/Alexa Fluor 488-labeled siRNA complexes (1 µM/100 nM) for 2, 4, 6, 8, 10, 12 and 24 h. Fluorescence intensity inside the cells was determined by selecting regions of interest. Data are expressed as the average ± SEM (*n* = 50).

Incubation of C6, GL261, U87, and T98G glioblastoma cell lines with AMC6/Alexa 488 Fluor-siRNA for different times showed a progressive increase in fluorescence up to 6 h (Figure 2). Then, intracellular fluorescence signal was stable up to 8 (C6 cells) or 12 h (GL261 and T98G). In the case of U87 the fluorescent signal remained stable up to 24 h (Figure 2). Raw fluorescence images can be observed in the Supplementary Information (Figure S1). Since all glioma/glioblastoma cell lines showed a similar behavior on Alexa 488 Fluor-siRNA uptake, we decided to perform all the experiments in T98G human glioblastoma cells. This cell line is the only one among the indicated cells that expresses the DNA-repairing enzyme O^6^-methylguanine–DNA methyltransferase (MGMT) that removes the alkylation caused by temozolomide, a first-line key drug in glioblastoma treatment [30]. The methylation state of the MGMT promoter is a prognostic factor of the patients’ response to this drug [31].

**Figure 3:**
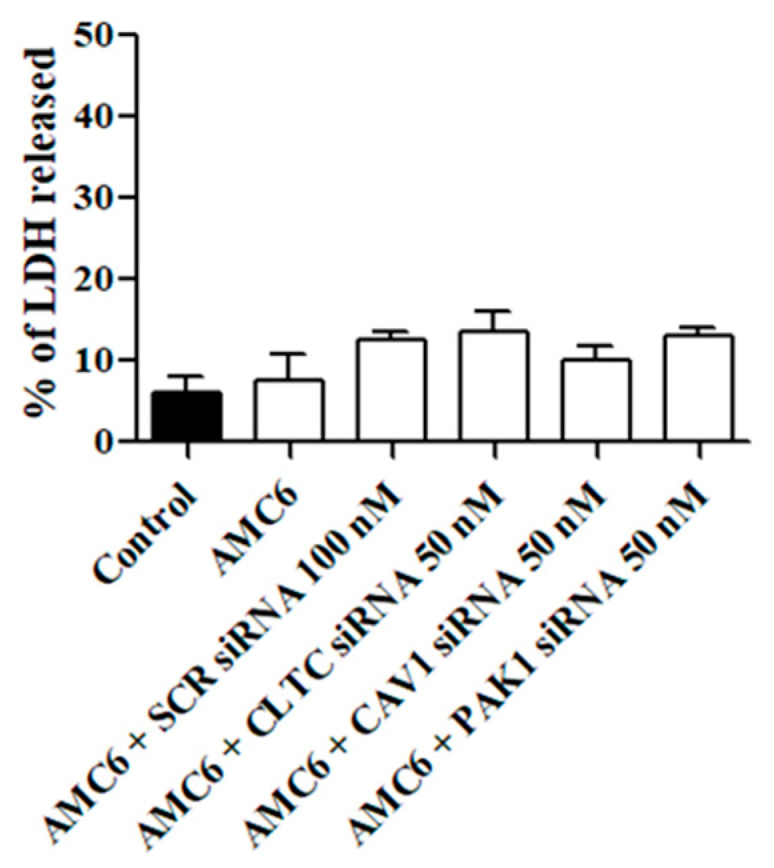
Effect of AMC6/custom siRNA against CLTC, CAV1 and PAK1 on cell viability. Toxicity is expressed as percentage of lactate dehydrogenase (LDH) released to the culture medium. Cells were treated with vehicle (Control), AMC6 (1 µM), AMC6 (1 µM) complexed with non-silencing scramble (SCR) siRNA (100 nM), and AMC6 (1 µM) complexed to 100 nM specific siRNA for CLTC, CAV1, and PAK1. Data are expressed as the mean ± SEM of 8 to 20 experiments.

Next, we studied whether the treatment with either the AMC6 vector alone or the nanoplexes was toxic for T98G glioblastoma cells. As it can be observed in Figure 3, AMC6 was not toxic by itself while nanoplexes formed by AMC6 plus either scramble (SCR) siRNA or plus one of the specific siRNAs only slightly increased toxicity in T98G cells (Figure 3). When SCR siRNA at a concentration of 50 nM was coupled to AMC6, LDH released amounted to 11.2 + 1.76% (*n* = 13).

### 2.2. Knockdown of Key Proteins Involved in Endocytic Pathways

**Figure 4:**
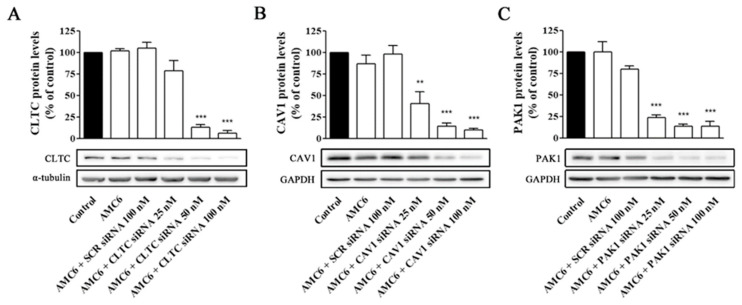
Knockdown of CLTC, CAV1 and PAK1 proteins by specific siRNAs. Cells were treated for 72 h with vehicle (Control), AMC6 (1 µM), AMC6 (1 µM) complexed with SCR siRNA (100 nM) or AMC6 (1 µM) complexed to increasing concentrations of specific siRNAs (25, 50 and 100 nM). The graph shows the ratios between the optical densities of CLTC and α-tubulin (**A**), CAV1 and glyceraldehyde 3-phosphate dehydrogenase (GAPDH) (**B**) and PAK1 and GAPDH (**C**), expressed as percentage in comparison to the vehicle (control). Western blot images belong to a representative experiment. Data are expressed as the mean ± SEM of 3 individual experiments. ** *p* < 0.01; *** *p* < 0.001 as compared to control.

We decided to study the effect of increasing concentrations of specific siRNA aimed to knockdown key proteins involved in CME (CLCT), CVME (CAV1), and macropinocytosis (PAK1). As it can be observed in Figure 4, neither AMC alone nor the AMC6/SCR siRNA (100 nM) nanoplexes significantly modified the target protein levels after 72 h of treatment (Figure 4).

**Figure 5:**
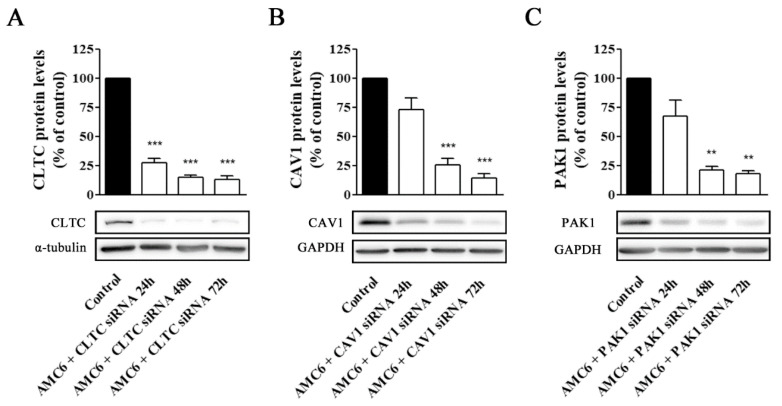
Time-course of AMC6/custom siRNA against CLTC, CAV1 and PAK1. Cells were treated with vehicle (control) for 72 h, and AMC6 (1 µM) complexed to CLTC (**A**), CAV1 (**B**) and PAK1 (**C**) targeting siRNA (50 nM) for 24, 48 and 72 h; The graph shows the ratios between the optical densities of CLTC and α-tubulin (**A**) CAV1 and GAPDH (**B**) and PAK1 and GAPDH (**C**), expressed as percentage in comparison to the vehicle (control). Western blot images belong to a representative experiment. Data are expressed as the mean ± SEM of 3 individual experiments. ** *p* < 0.01; *** *p* < 0.001 as compared to control.

In contrast, nanoplexes formed using specific siRNA at 50 and 100 nM concentrations knocked down the target protein levels by more than 80% (Figure 4). From these data, 50 nM siRNA was selected as the lowest siRNA concentration providing a very significant (more than 80%) reduction in target protein levels, which would allow studying the effect of lack-of-function for the target protein on the cell uptake efficiency of the nanocomplexes. No specific siRNA-mediated cross inhibition was observed with the other two proteins studied (supporting information, Figure S2). As it can be observed in Figure 5, a significant protein knockdown, allowing lack-of-function studies, could be achieved at 48 h for the three studied proteins. Thus, this time point was also selected for future experiments.

### 2.3. Effect of Signaling Protein Knockdown on Function of Endocytic Mechanisms

Once optimal conditions for efficient knockdown of the target proteins were established, we tested whether removal of the function of these proteins was able to block endocytosis through the involved endocytic pathway. To explore this, we used Alexa 555-labelled human transferrin that is taken up selectively by a CME [32] and tetramethylrhodamine (TMR)-labelled 70 kDa dextran which is specifically endocytosed by macropinocytosis [33]. No compound has been described yet to be specifically taken by the caveolin-dependent pathway, although cholera toxin is taken preferentially, but not exclusively by this pathway [34].

**Figure 6:**
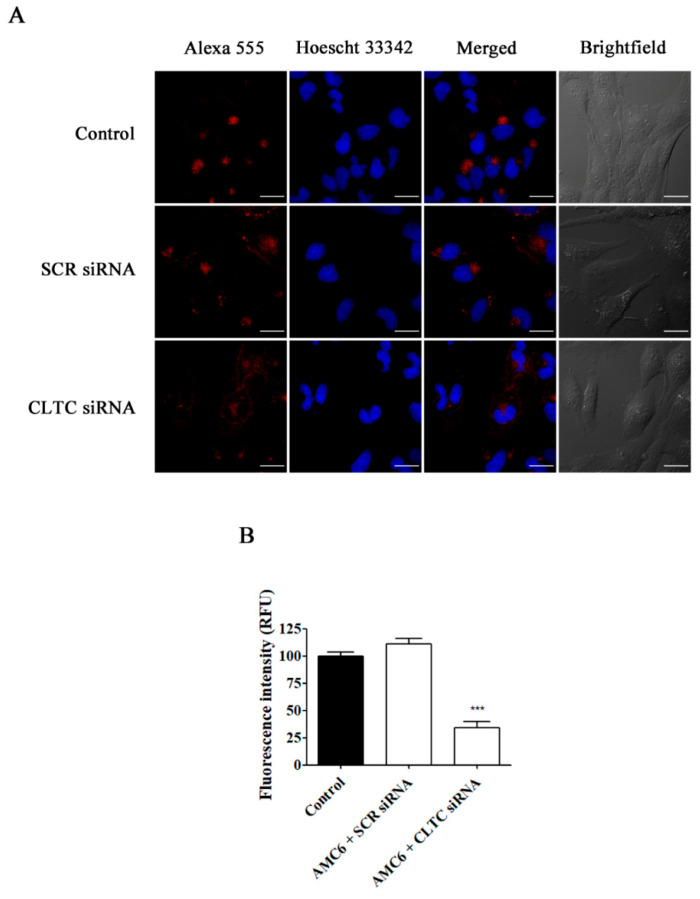
Effect of the downregulation of CLTC on the uptake of Alexa Fluor 555 conjugated transferrin. T98G cells were pre-treated with vehicle (control), AMC6/SCR siRNA and AMC6/anti-CLTC siRNA complexes (both 1 µM/50 nM) for 48 h to be later treated for 30 min with fluorescent Alexa Fluor 555 labeled human transferrin (25 µg/mL). The images panel (**A**) shows Alexa Fluor 555-transferrin (red), Hoescht 33,342 (blue) and the overlay of both images. In addition, differential interference contrast (DIC) images are also shown. Scale bar: 25 µm; (**B**) Quantification of fluorescence (expressed in Relative Fluorescence Units; RFU) for the 3 experimental conditions. Data are expressed as the mean ± SEM (*n* = 50). *** *p* < 0.001, compared to control cells.

**Figure 7:**
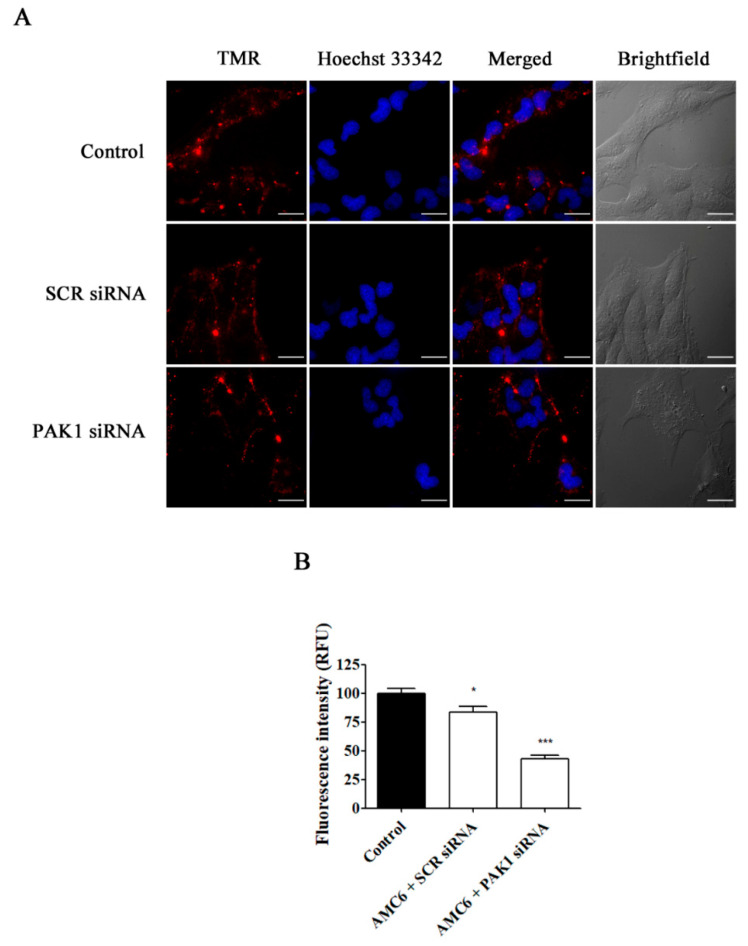
Effect of the downregulation of PAK1 in the uptake of TMR-conjugated 70 kDa dextran. T98G cells were pre-treated with vehicle (control), AMC6/SCR siRNA and AMC6/anti PAK1 siRNA (both 1 µM/50 nM) for 48 h to be later treated for 1 h with TMR-labeled 70 kDa dextran (0.5/mL). The images panel (**A**) shows TMR-70 kDa dextran (red), Hoescht 33,342 (blue) and the overlay of both images). In addition, differential interference contrast (DIC) images are also shown. Scale bars: 25 µm; (**B**) Quantification of fluorescence (expressed in RFU) for the 3 experimental conditions. Data are expressed as the mean ± SEM (*n* = 50).* *p* < 0.05, *** *p* < 0.001, compared to control cells.

As it can be observed in Figure 6, knockdown of CLTC for 48 h decreased transferrin uptake by about 70%, while nanoplexes formed by AMC6 and SCR siRNA (50 nM) lacked any effect. Moreover, when PAK1 was knocked down, TMR-labeled 70 kDa dextran uptake was decreased by almost 60% (Figure 7). In this case, nanoplexes formed by AMC6 and SCR slightly decreased TMR-70 kDa dextran uptake (about 15%) indicating a small unspecific effect of siRNA on this endocytic pathway.

### 2.4. Effect of Selective Blockade of Endocytic Pathways on AMC6/siRNA Nanoplexes Uptake

To study whether the selective blockade of a single endocytic pathway (CME, CVME or macropinocytosis) could lead to a marked decrease in AMC6-mediated FAM-siRNA uptake, we treated T98G glioblastoma cells with nanoplexes formed by AMC6 and siRNA, either SCR or aimed to knockdown CLTC, CAV1 or PAK1, for 48 h. At that time, we washed the cells with culture medium to remove non-taken up nanoplexes. Then, new nanoplexes formed by AMC6 and Alexa 488 Fluor-siRNA were added to assess siRNA uptake.

**Figure 8:**
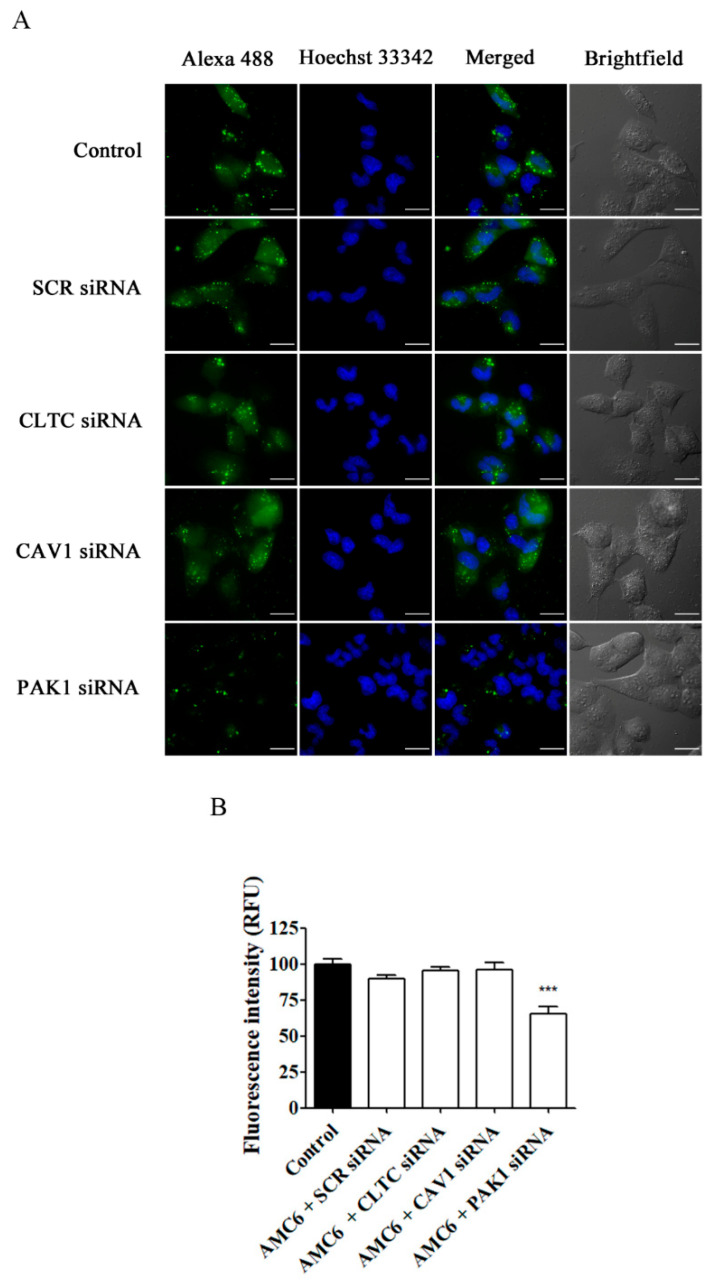
Effect of knocking down key proteins in CME, CVME and macropinocytosis on the uptake of AMC6/Alexa Fluor 488-labelled siRNA. T98G glioblastoma cells were pre-treated for 48 h with vehicle (control) and AMC6/siRNA complexes (1 µM/50 nM) consisting in SCR siRNA, or specific siRNAs targeting CLTC, CAV1, and PAK1. After this, cells were treated for 6 h with AMC6/alexa488 siRNA (green) complexes (1 µM/100 nM) and with Hoechst 33,342 (25µ/mL) (blue) for 10 min before image recording. (**A**) The images panel (**A**) shows Alexa Fluor 488-siRNA, Hoescht 33,342 and the overlay of both images). In addition, DIC images are also shown. Scale bars: 25 µm; (**B**) Quantification of fluorescence (expressed in RFU) for all the experimental conditions. Data are expressed as the mean ± SEM (*n* = 100). *** *p* < 0.001, compared to control cells.

As it can be observed in Figure 8, only knocking down PAK1, implying macropinocytosis inhibition (Figure 7), reduced the cellular uptake of Alexa 488 Fluor-siRNA by 35%, suggesting that the non-blocked uptake pathways cannot fully compensate for macropinocytosis blockade. On the other hand, knocking down either CLTC or CAV1 did not decrease AMC6-mediated Alexa 488 Fluor-siRNA uptake, suggesting that either these endocytic routes are not significantly involved in the internalization of the nanocomplexes or that they can be compensated by the other non-blocked endocytic pathways.

### 2.5. Effect of Specific Blockade of siRNA Uptake Pathways on Transfection Efficiency

Once we established that we could selectively and efficiently block either CME, CVME or macropinocytosis, we explored whether the decrease in AMC6-mediated Alexa Fluor 488-labelled siRNA cellular uptake following the observed knockdown of PAK1 and macropinocytosis blockade was correlated with a decrease in AMC6 transfection ability. For this purpose, we first selectively knocked down either PAK1, CLTC or CAV1 (the latter used as controls) by incubating the cells with 50 nM of the specific siRNA (50 nM) for 48 h. At that time, target proteins levels were decreased to about 20% of their initial values (Figure 5) and the corresponding pathways blocked (Figure 6 and Figure 7).

**Figure 9:**
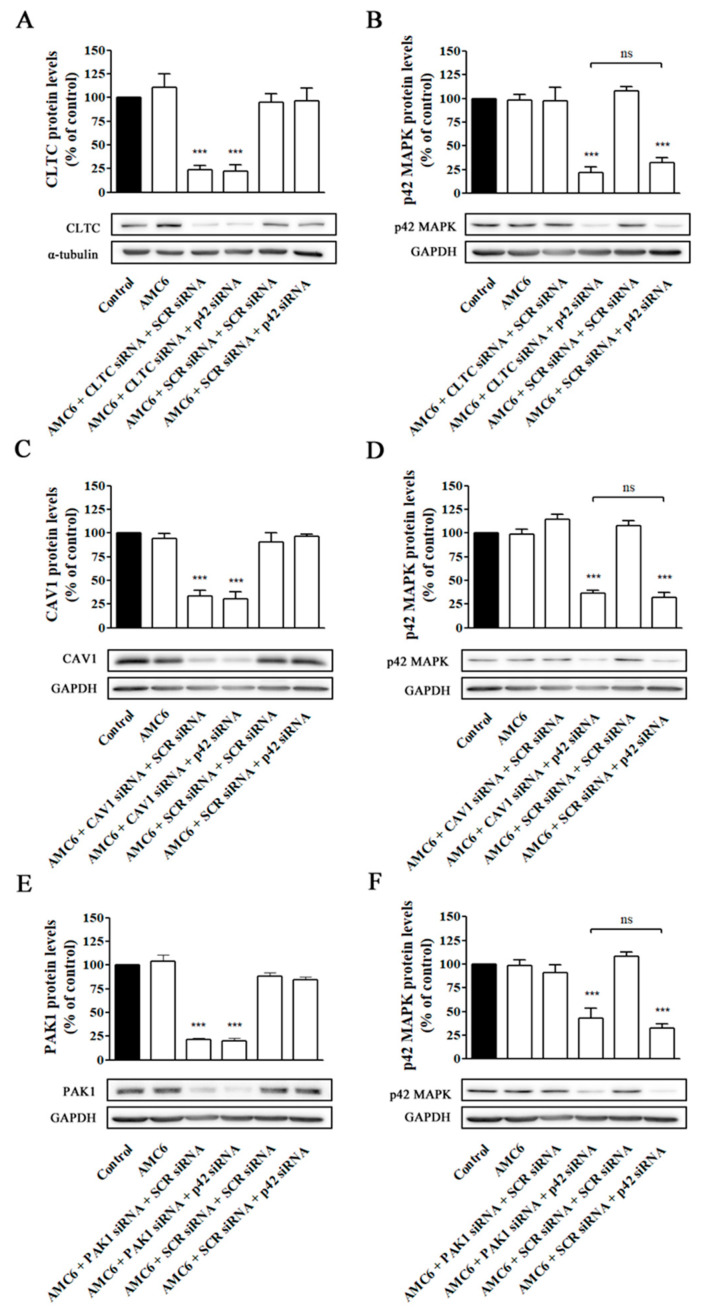
Effect of selective knockdown of CLTC (**A**,**B**), CAV1 (**C**,**D**), and PAK1(**E**,**F**) on AMC6-mediated transfection efficiency. T98G cells were treated for 48 h with either vehicle (control), AMC6 (1 µM) or nanoplexes formed by AMC6 (1 uM)/specific siRNA (50 nM) targeting CLTC (**A**), CAV1 (**C**) and PAK1 (**E**). Then, the culture medium was replaced by one containing nanoplexes formed by AMC6 and 50 nM of either SCR siRNA or anti-p42- mitogen-activated protein kinase (p42 MAPK) and cells were incubated for additional 48 h. CLTC (**A**), CAV1 (**C**), and PAK1 (**E**) protein levels were determined respect to control values. In the experiments shown in panels B, D, and F, the cells received the same treatments, but p42-MAPK levels were determined instead. Western blot images shown representative experiments for each protocol. Data are expressed as mean ± SEM of 3 or 4 individual experiments for each group. *** *p* < 0.001 as compared to control; ns, statistically non-significant.

Then, the medium was changed to remove non-taken nanoplexes and new medium containing nanoplexes formed by either AMC6 (1 µM) plus either SCR siRNA (50 nM) or anti-p42 mitogen-activated protein kinase (p42 MAPK) siRNA (50 nM) was added and incubation continued for additional 48 h. This anti-p42 MAPK siRNA has shown previously that is able to reduce p42 MAPK protein levels to about 20% of control values [29]. As it can be observed in Figure 9, blockade of one single pathway does not prevent βCD-based nanoplexes to efficiently knockdown p42 MAPK protein levels, suggesting that, in functional terms, AMC6/siRNA nanocomplexes are taken up by the three major endocytic uptake pathways that compensate among them, maintaining a high level of siRNA transfection if one of those mechanisms is blocked.

## 3. Discussion

siRNA has established itself as a new tool for exploring the role that certain proteins have in both the pathogenesis of certain diseases and in dissecting signaling pathways in different systems. The high specificity and effectivity of siRNA for knocking down target proteins has prompted its use as therapeutics for a number of diseases [6,7]. siRNA is a labile molecule and so it requires appropriate carrier systems (vectors) to protect it from RNAse-mediated degradation [35] and to transport it to the cell interior, where it might act as a therapeutic molecule knocking down the target protein. NP-forming vectors offer an additional advantage since their outer peripheral groups can be modified to specifically delivery the NP and its cargo to the target cell [36].

βCD is a highly biocompatible carbohydrate-derivative compound that has been approved for use in humans [37]. βCD-based nanostructures have demonstrated a great potential as genetic material transfection vectors [38]. In fact, the first siRNA clinical trial that utilized a targeted NP delivery system (CALAA-01) used a cyclodextrin [39]. AMC6 is a strictly monodisperse polycationic amphiphilic βCD derivative with the capability to co-assemble with siRNA to form nanocomplexes that have shown a high siRNA transfection efficiency in several tumoral cell lines from different species (human, rat, mouse) [29].

The chemical characteristics that a NP should have to become an efficient siRNA transfection vector remain elusive and most of the approaches to elucidate this issue are based on trial and error tests. Molecular vectors with perfectly monodisperse architectures based on rigid nanometric platforms, allowing systematic structural variations to optimize nucleic acid complexation and self-assembly through precision chemistry, are notable exceptions [40,41]. Indeed, AMC6 was selected as a privileged compound for siRNA delivery on the basis of structure–activity relationship studies. Another relevant factor that might influence siRNA transfection efficiency is the uptake pathway followed by the NP and its siRNA cargo to enter the cell. The vast majority of nanoparticles get access to the cell interior through endocytosis [13], mainly through three main endocytic pathways: macropinocytosis [42], CME [43], and CVME [44]. The compounds taken up by these endocytic pathways have a common final fate in the lysosomes. A key step in obtaining high transfection efficiency is the timely endosomal escape of the nanoparticle and its cargo to avoid the lytic environment of lysosomes [45]. Other endocytic mechanisms have been described in different cell types such as adenosine diphosphate-ribosylation factor 6 (Arf-6) [46], GRAF1, Flotillin [47], RhoA or CDC-42 mediated endocytosis. However, its contribution to NP uptake into the cells is very minor.

An unresolved question is whether or not the endocytic pathway followed by a siRNA-loaded NP markedly influences the transfection efficiency. If affirmative, it would lead to specifically designed NPs that would be preferentially taken up by the most efficient route. This has been initially explored using different pharmacological inhibitors for each pathway such as chlorpromazine (CME) [18,19], genistein [48] (CVME), and amiloride hydrochloride (macropinocytosis) [49] among others. However, the lack of specificity and the simultaneous blockade of several pathways by the same drug have precluded to obtain clear-cut results [21]. To overcome these limitations, we decided to take advantage of the high transfection efficiency of the βCD-derivative AMC6 to selectively knockdown CLTC, CAV1 and PAK1, which are key proteins in CME, CVME and macropinocytosis, respectively. We aimed at selectively blocking each of those pathways individually in order to explore their contribution to efficient AMC6-mediated transfection efficiency. The CLTC protein is critical for the formation of the clathrin lattice, and its knockdown selectively blocks CME without interfering with the uptake of specific markers for other pathways such as the 70 kDa dextran, a marker for macropinocytosis [33]. CAV1 is responsible for the formation and structural stability of caveolae in CVME [50]; it does not co-localize with transferrin [51] and its knockdown does not alter transferrin uptake [52], suggesting a high selectivity for CVME. Finally, PAK1 knockdown interrupts the route of actin polymerization after the activation of both Rac1 and Cdc42 by Ras, and impedes macropinosomes closure through CtBP1/BARS and myosin [24].

AMC6-Alexa 488 Fluor-siRNA complexes were quickly taken up by different human (U87 and T98G), rat (C6), and mouse (GL261) glioblastoma cell lines, indicating the ability of the NPs to deliver siRNA to target cells [29]. We decided to use the human T98G cell line as the experimental model for further studies based on its human origin and the high expression levels of MGMT, whose gene methylation is a marker for better prognosis of glioblastoma patients [53]. Nanocomplexes formed by AMC6 plus specific siRNAs were able to reduce cellular protein levels of either CLTC, CAV1 or PAK1 to about 20 to 25% of control values, enabling lack-of-function studies. This blockade remained stable up to 96 h after the initial treatment. Under these experimental conditions, it was observed that the individual blockade of CME or CVME did not reduce AMC6-mediated fluorescent siRNA uptake by T98G human glioblastoma cells, suggesting that either those pathways do not participate in AMC6-mediated siRNA uptake or the unblocked endocytic pathways compensate for the blockade of a single individual pathway. On the other hand, blockade of macropinocytosis by knocking down PAK1 reduced siRNA AMC6-mediated siRNA uptake by 35%, indicating that macropinocytosis is the major mechanism for AMC6-siRNA nanocomplex uptake into glioblastoma cells and that the combined action of the other two major endocytic pathways cannot fully compensate for macropinocytosis blockade. Notwithstanding, the blockade of either individual pathway (CME, CVME or macropinocytosis) did not significantly reduced the ability of AMC6 to efficiently deliver a specific siRNA aimed to reduce intracellular p42 MAPK protein levels. This result suggests that besides not being able to fully compensate for the loss of AMC6-mediated siRNA entry when macropinocytosis is blocked, the amount of siRNA taken up by the cells through other active endocytic mechanisms (CME and CVME) is efficient enough to knockdown p42 MAPK to about 25% of the control values.

Macropinocytosis is a highly conserved endocytic process whereby extracellular fluid and its contents are internalized into cells through large, heterogeneous vesicles known as macropinosomes. The fact that macropinocytosis is the main mechanism for siRNA uptake in T98G cells might represent an additional advantage for treating glioblastoma [54]. Indeed, macropinocytosis is stimulated in tumoral cells, where it represents an adaptative mechanism to increase the uptake of extracellular proteins and other nutrients to address their increased metabolic demands [55] as well as to contribute to other processes such a signal transduction or membrane surface receptor regulation [56] and metastasis [57]. Targeting NPs and their therapeutic cargo to be taken up by macropinocytosis would increase the therapeutic effectivity of the cargo molecule. This approach has been attempted by conjugation of the NP to keratinocyte growth factor receptors [58] or to the collagen receptor α2β1 integrin, whose internalization is also mediated by macropinocytosis [59]. However, a more interesting strategy consists of developing compounds that, by their own characteristics, are directly taken up by macropinocytosis by the tumoral cells. The βCD derivative AMC6 belongs to this group of compounds since no modifications with specific ligands are needed to preferentially enter the cells by macropinocytosis.

In summary, here we have shown the high transfection ability of a βCD-derivative, AMC6, that is able to efficiently deliver siRNA to different glioblastoma cell lines. Moreover, delivery by AMC6 of specific siRNA aimed to knockdown key proteins (CLTC, CAV1, and PAK1) involved in the major physiological endocytic mechanisms CME, CVME and macropinocytosis, respectively, can selectively block the target endocytic pathway. The results presented here show that siRNA is an excellent tool to dissect pathophysiological mechanisms and that, due to its great selectivity, it markedly avoids possible problems of interpretation derived from the lack of specificity of many pharmacological tools. Moreover, the fact that macropinocytosis is the main gateway for AMC6-mediated siRNA uptake suggests that targeting these uptake mechanisms by NPs might increase the delivery of therapeutic molecules to tumoral cells and, thus, enhancing the therapeutic efficiency of them and their cargo.

## 4. Materials and Methods

### 4.1. Cell Culture

Several glioma/glioblastoma cell lines from different species (C6, rat glioma; U87 human glioblastoma; T98G human glioblastoma) were obtained from ATCC (Manassas, VA, USA). The GL261 mouse glioma was obtained from Leibniz-Institut DSMZ (Braunschweig, Germany). All cell lines were cultured according to the provider’s instructions. The culture medium consisted in high glucose Dulbecco’s Modified Eagle’s Medium (DMEM) supplemented with 10% heat-inactivated FBS; 2 mM L-glutamine, 5 μg/mL streptomycin and 20 units/mL penicillin. Cells were incubated for 24 h at 37 °C under a humidified atmosphere with 5% CO_2_ in the case of C6, U87, and T98G, and 10% in the case of GL261.

### 4.2. Fluorescent siRNA Uptake

T98G human glioblastoma cells were seeded on glass coverslips of 20 mm diameter at a concentration of 100,000 cells/mL in 6 well plates containing 1 mL of culture medium and incubated for 24 h under the above indicated conditions. Then, the coverslips were washed 3 times with Krebs-Henseleit (K-H) solution with the following ionic composition (in mM): NaCl, 140; CaCl_2_, 2.5; MgCl_2_, 1 KCl, 5; Hepes 5, Glucose, 11 (pH, 7.4) and treated with the AMC6/Alexa Fluor 488 fluorescent siRNA (FAM-siRNA) complexes (1 µM/100 nM) for the indicated times. In some experiments, 10 min before the recording time, Hoescht 33,342 (25 µg/mL) (ThermoFisher, Waltham, MA, USA) was added to label nuclei. Following the incubation time, cells were washed 3 times with K-H solution and mounted on the stage of a Nikon Eclipse TE2000-E fluorescence microscope (Nikkon, Tokyo, Japan).

Once the cells were treated, fluorescence was recorded using 40× or 60× fluorescence oil immersion objectives. Excitation wavelengths were 350 nm for Hoechst 33,342 and 488 nm for Alexa Fluor 488 while emission wavelengths were 450 nm for Hoechst 33,342 and 520 nm for Alexa Fluor 488. Data were acquired using NIS Elements AR software (Nikkon, Tokyo, Japan). Image analysis was performed using Image J software (v1.53f, NIH, Bethesda, MD, USA) [60]. Regions of interest (ROIs) corresponding to individual cells were selected and the fluorescence intensity of the pixels present in that area measured in RFU. Transfection index was calculated considering as positive cells those with more than 2000 RFU.

### 4.3. Protein Knock Down

Cells were seeded in p24 plates (Corning, NY, USA) at 50,000 cells/mL 24 h before starting the experiment. Then, cells were incubated for the indicated times with AMC6 (1 µM) alone or with nanoplexes formed by AMC6 (1 µM) plus either scramble siRNA (SCR; 100 nM; Sigma, Barcelona, Spain) or the desired concentration of specific siRNA aimed to knockdown CTLC, CAV1, PAK1 or p42 MAPK for different times. After the incubation period, cells were washed 3 times in K-H medium (see SM for the ionic composition), lysed, protein content determined and samples processed for western blot as previously described [61]. siRNAs were custom-designed and directed against mRNA sequences 613-625 (CLTC), 643-661 (CAV1), and 667-699 (PAK1).

### 4.4. Western Blot

Western blots were performed as previously described [62] using PAGE-SDS gels of 8% for CLTC, 10% for PAK1, 12% for p42 Mitogen-Activated Protein Kinase (p42-MAPK) and 15% for CAV1. Twenty-five µg of protein were loaded into each lane. The following polyclonal antibodies, all purchased from Cell Signaling (Danvers, MA, USA), were used: anti-CLTC (1:1000); anti-α-tubulin (1:2000); anti-CAV1 (1:1000); anti-GAPDH (1:2000); anti-PAK1 (1:1000); and anti-p42-MAPK (1:1000). GAPDH and α-tubulin were used as loading control indicators. Immunocomplexes were visualized using an enhanced chemiluminescence system (Millipore, MA, USA) and densitometric analysis of immunoreactive bands was performed using the Image J program [60].

### 4.5. Cell Viability Assays

Cell viability was determined by measuring Lactate dehydrogenase (LDH) release as an index of cellular death as previously described [11]. Cells were seeded in p24 plates (Corning, NY, USA) at 50,000 cells/mL and incubated for 24 h. Following the different treatments, cell culture media were collected, and the cells, attached to the plate, washed with phosphate buffered saline (PBS) and lysed using Triton X-100 (0.9% *v/v*) in PBS. After this step, LDH enzymatic activity, both in culture medium and in cell lysates, was measured using the Kit CytoTox 96^®^ (Promega, Madison, WI, USA) in a spectrophotometer at 490 nm wavelength. Toxicity was expressed as the percentage of LDH released to the culture medium respect to total LDH present in the cells at the beginning of the experiment (total LDH present in cell lysates plus LDH present in the culture medium).

### 4.6. Specific Blockade of Major Endocytosis Pathways

Cells were seeded on 20 mm diameter glass coverslips at a concentration of 50,000 cells/mL. Twenty-four hours later, cells were treated with nanoplexes formed by AMC6 (1 µM) and specific siRNA (50 nM) aimed to knockdown key proteins involved in each particular endocytic pathway for 48 h. After that time, culture medium was removed and the cells were washed twice in sterile PBS. The medium was changed to K-H solution containing either transferrin from human serum conjugated to Alexa Fluor 555 (25 µg/mL; 30 min) (Thermo Fisher Scientific, Waltham, MA, USA), whose uptake is specific of CME [32], or 70 kDa dextran conjugated with TMR (0.5 mg/mL; 1 h) (Thermo Fisher Scientific Waltham, MA, USA), whose uptake takes place specifically by macropinocytosis [33]. After the incubation period, cells were washed twice with PBS, the coverslips were mounted on the stage of a Nikon Eclipse TE2000-E fluorescence microscope (Nikkon, Tokyo, Japan) and fluorescent images acquired using the NIS Elements AR software (Nikkon, Tokyo, Japan) as indicated above. Image analysis was performed using Image J software [60].

### 4.7. Statistical Analysis

Data are expressed as mean ± SEM Statistical analyses was performed using one-way analysis of variance and later a Tukey test for comparison of multiple columns by using GraphPad software (GraphPad, San Diego, CA, USA). p values smaller than 0.05 were considered significant (* *p* < 0.05, ** *p* < 0.01, *** *p* < 0.001). Statistical significances are reported in the figure legends.

## Supplementary Materials

The following are available online at https://www.mdpi.com/1422-0067/21/23/9306/s1.

## Abbreviations

βCD	β-cyclodextrin
CLTC	Clathrin Heavy Chain 1
CME	Clathrin mediated endocytosis
CVME	Caveolin mediated endocytosis
GAPDH	Glyceraldehyde 3-phosphate dehydrogenase
HIV	Human immunodeficiency virus
K-H	Krebs-Henseleit
LDH	Lactate dehydrogenase
MGMT	O^6^-methylguanine–DNA methyltransferase
NP	Nanoparticle
PAK1	P21 (RAC1) activated kinase 1
p42 MAPK	p42 MAP kinase
PBS	Phosphate buffered saline
RFU	Relative fluorescence units
RNA	Ribonucleic acid
RNAi	RNA interference
ROI	Region of interest
SCR	Scrambled
SEM	Standard error of the mean
siRNA	Small interfering RNA
TMR	Tetramethylrhodamine
