# Supplementary material for: Cyclodextrin-Based Nanostructure Efficiently Delivers siRNA to Glioblastoma Cells Preferentially via Macropinocytosis
Source: Int J Mol Sci. 2020 Dec 6;21(23):9306. doi: 10.3390/ijms21239306 (PMC7731237; doi:10.3390/ijms21239306)
Supplement: Supplementary file 1 [file ijms-21-09306-s001.pdf]

International Journal of
Molecular Sciences

MDPI

Supporting Information

Figure S1. AMC6/Alexa 488 Fluor siRNA complexes uptake time course.

C6 cells.

2h

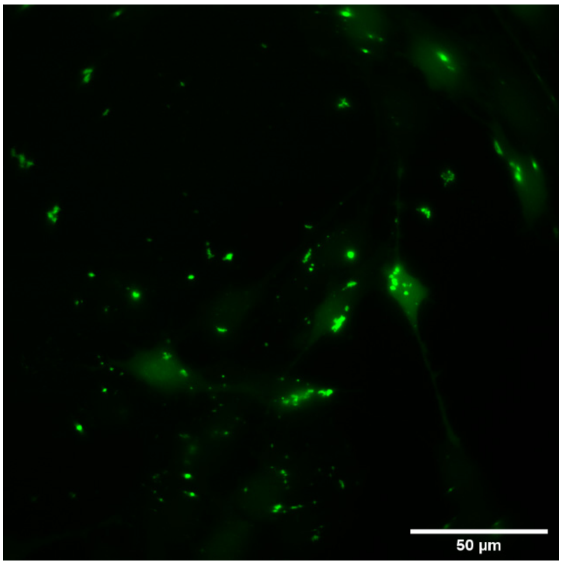

4h

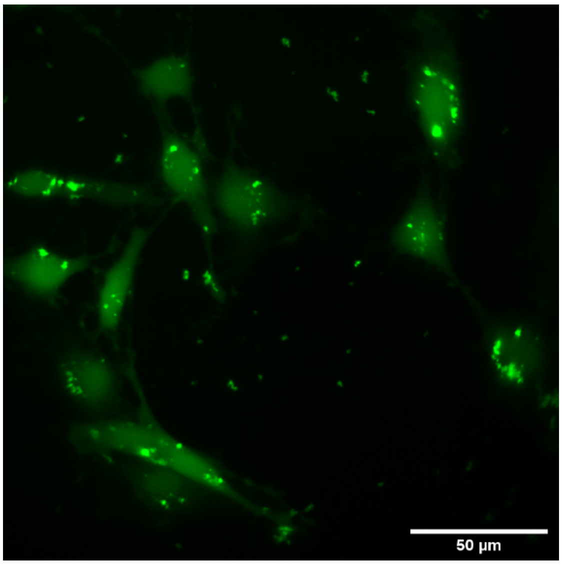

6h

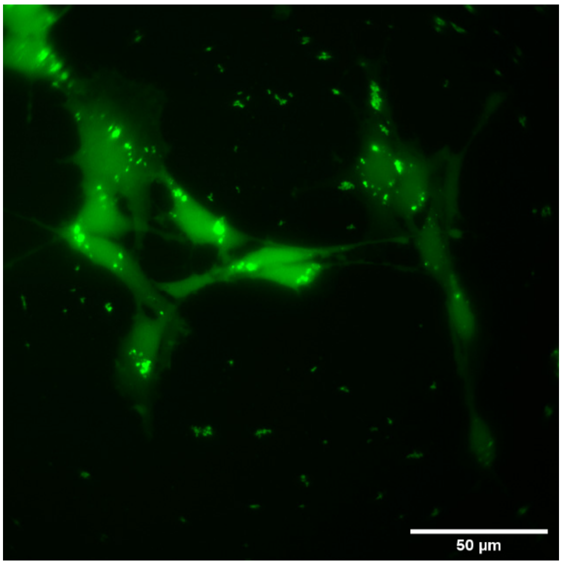

8h

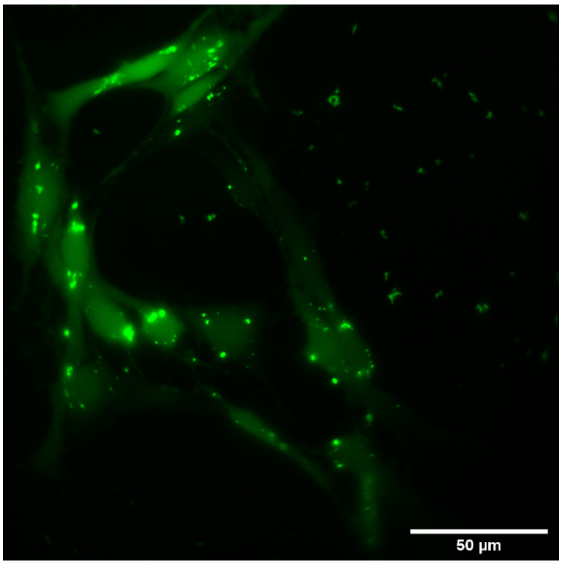

10h

12h

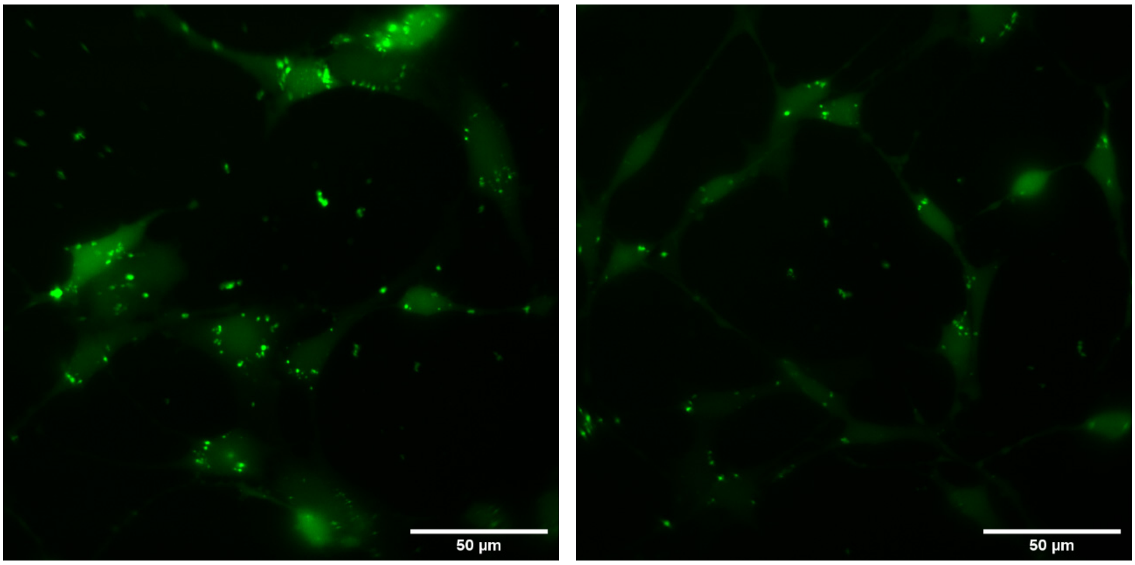

24h

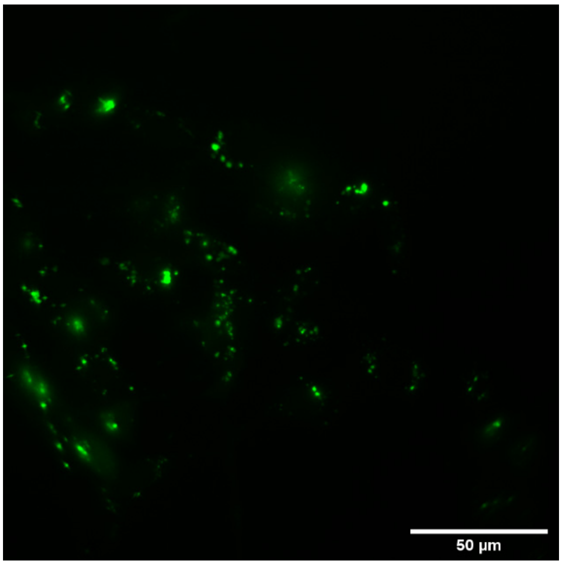

U87 cells

2h

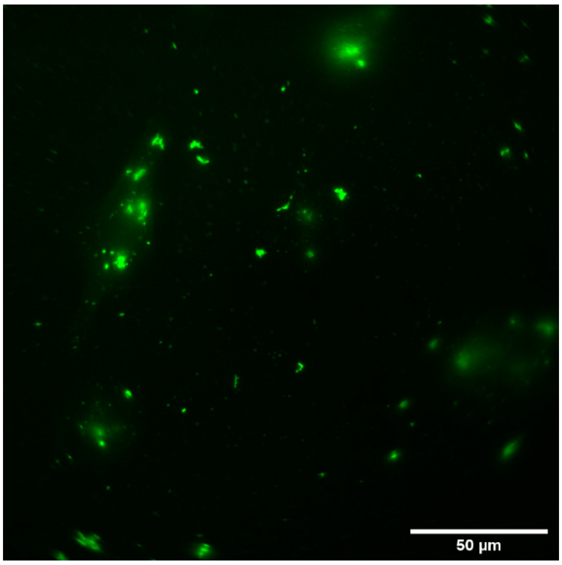

4h

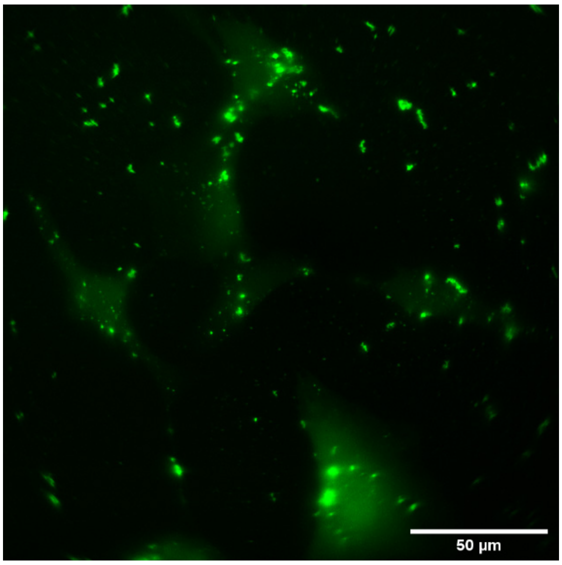

6h

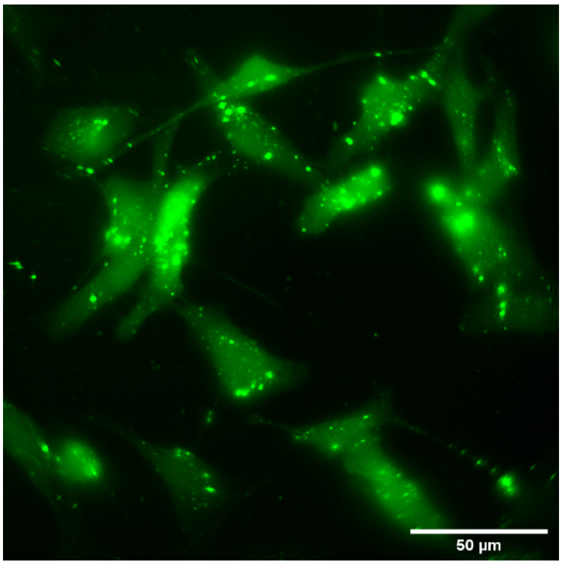

8h

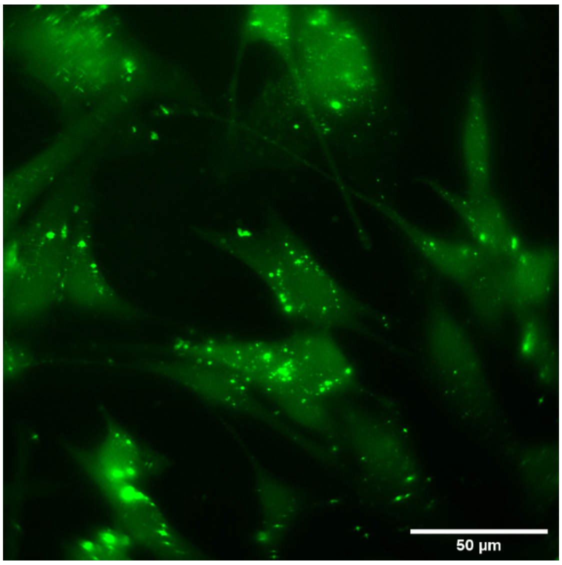

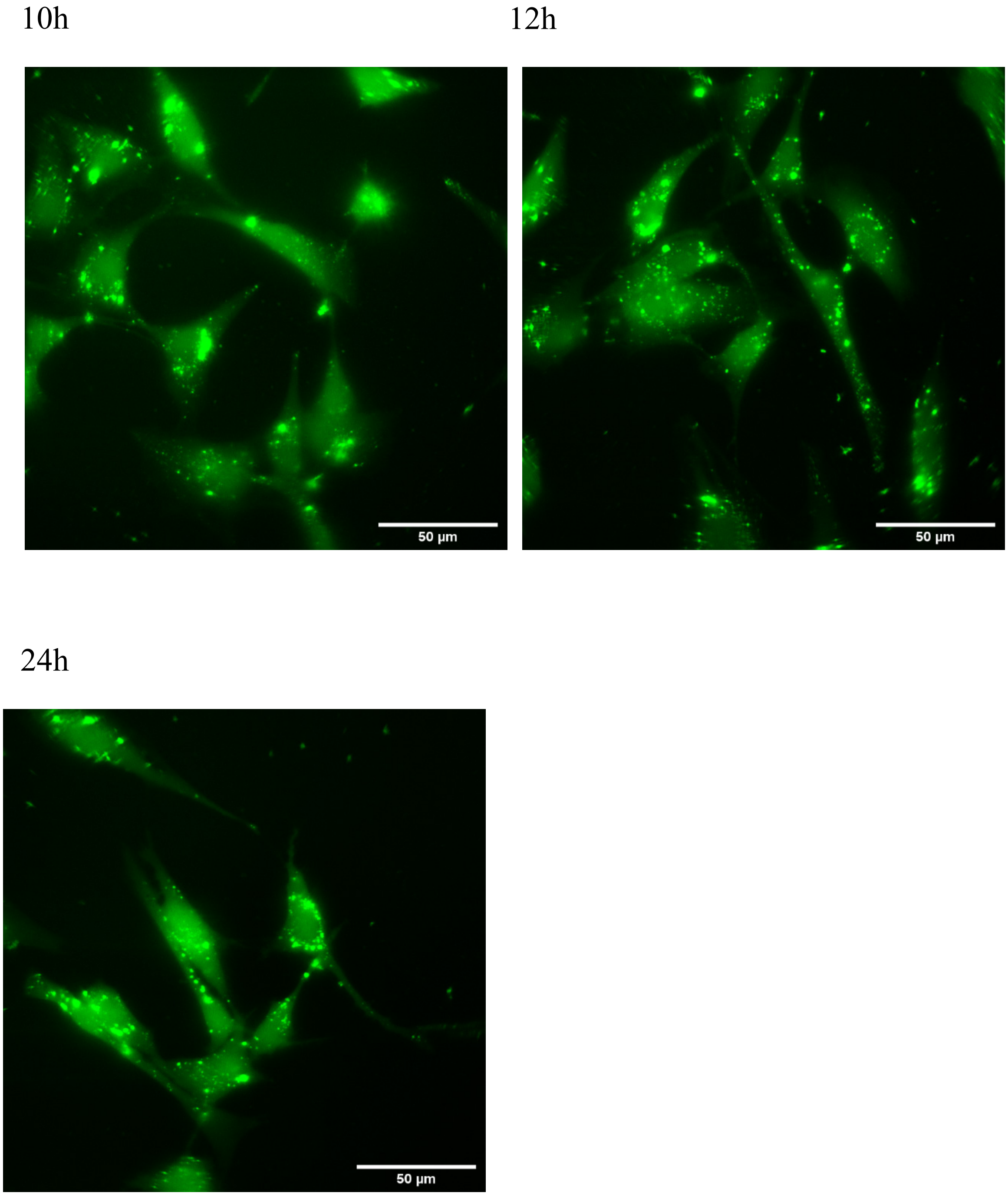
10h
12h
50 µm
50 µm
24h
50 µm

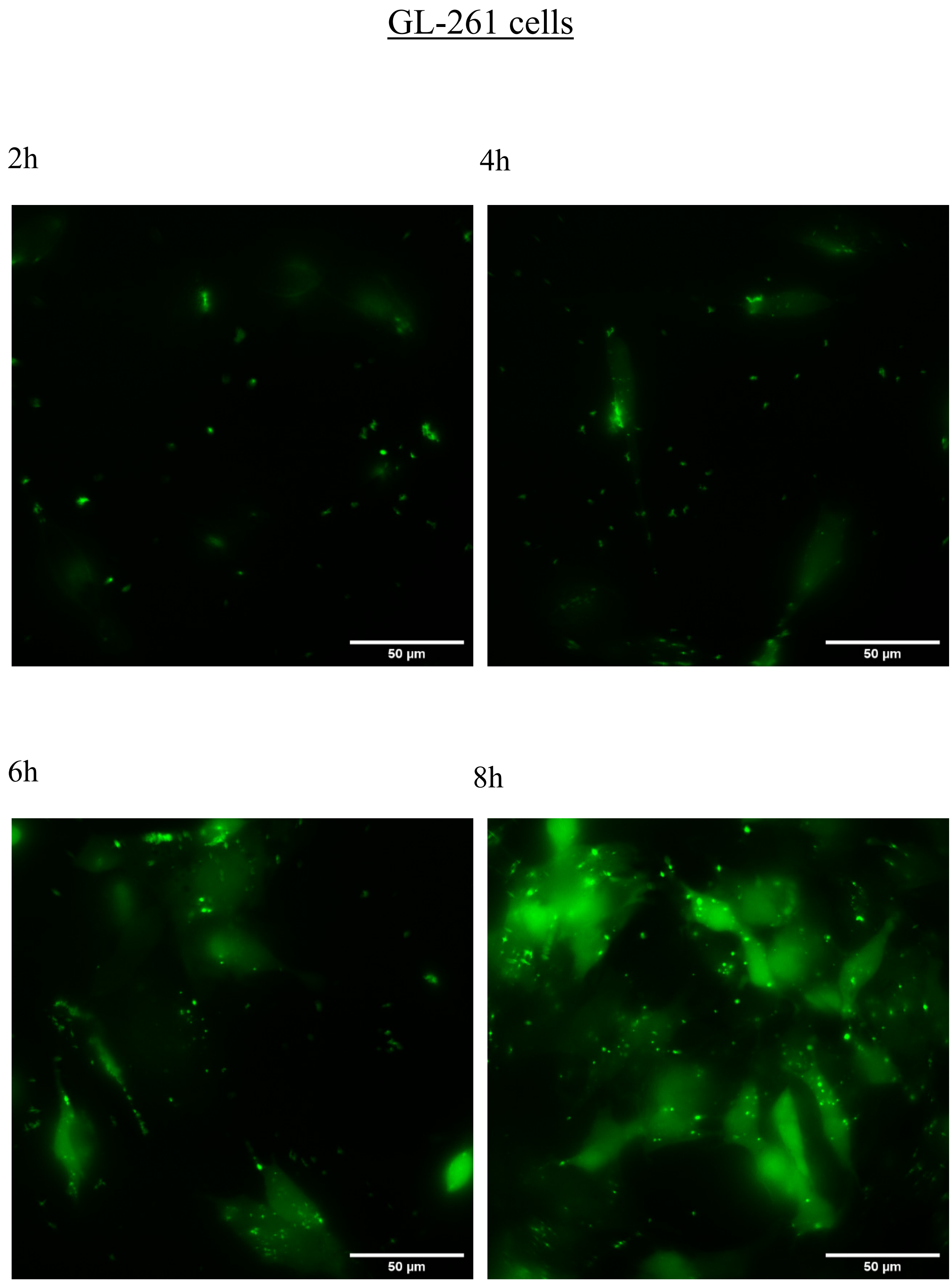
GL-261 cells
2h
4h
6h
8h
50 µm
50 µm
50 µm
50 µm

10h

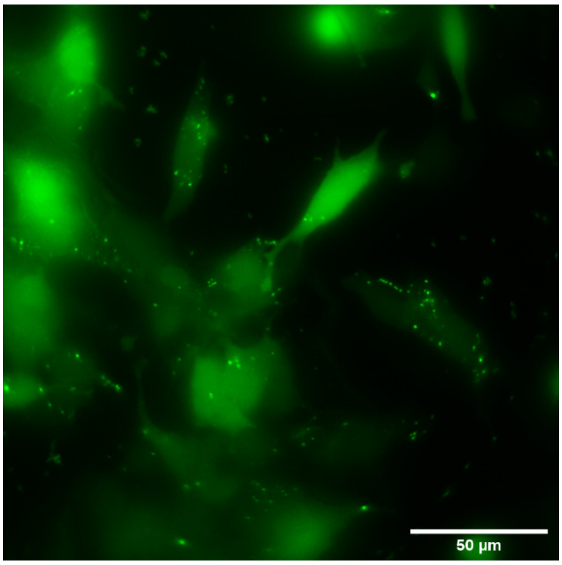

12h

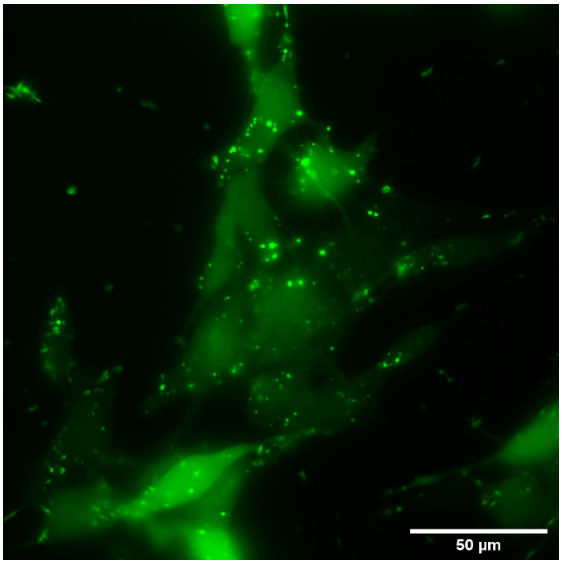

24h

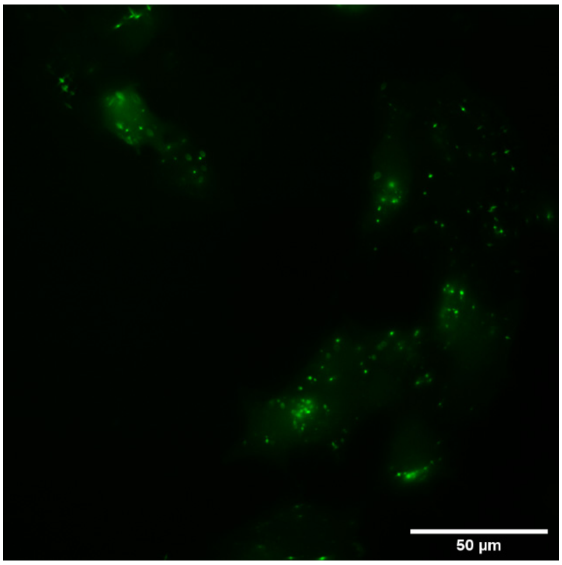

T98G cells

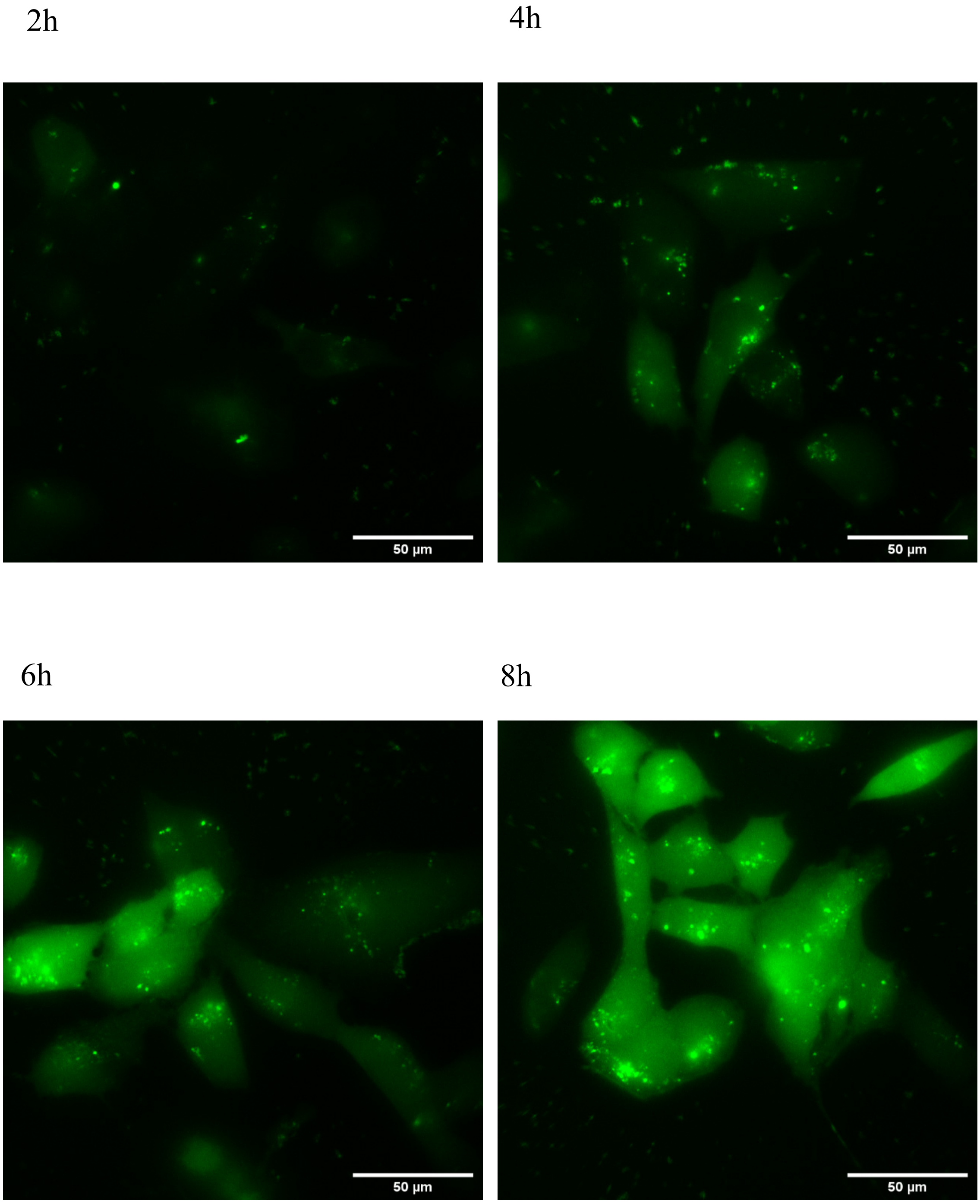

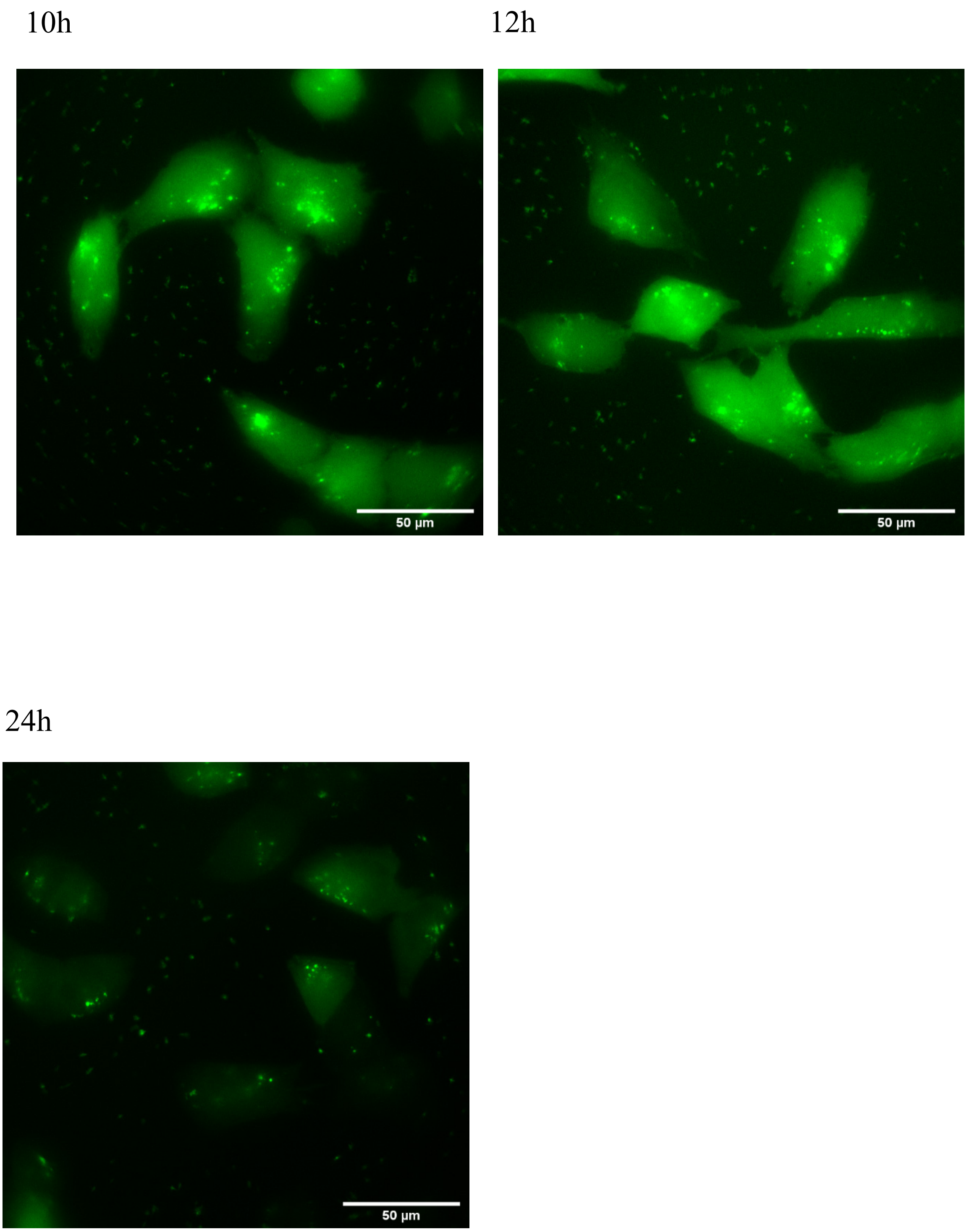
10h
12h
50 µm
50 µm
24h
50 µm

Figure S2. Effect of CLTC, CAV1 or PAK1 knock-down on the other endocytosis proteins. Specific down-regulation (with AMC6 complexed to the corresponding siRNA at 50 nM) of any of the key endocytosis proteins studied does not affect cellular levels of the non-targeted proteins. α-Tubulin was used as loading control for CLTC, while GAPDH was used in the case of CAV1 and PAK1. A. CLTC knock-down. B. CAV1 knock-down. C. PAK1 knock-down.

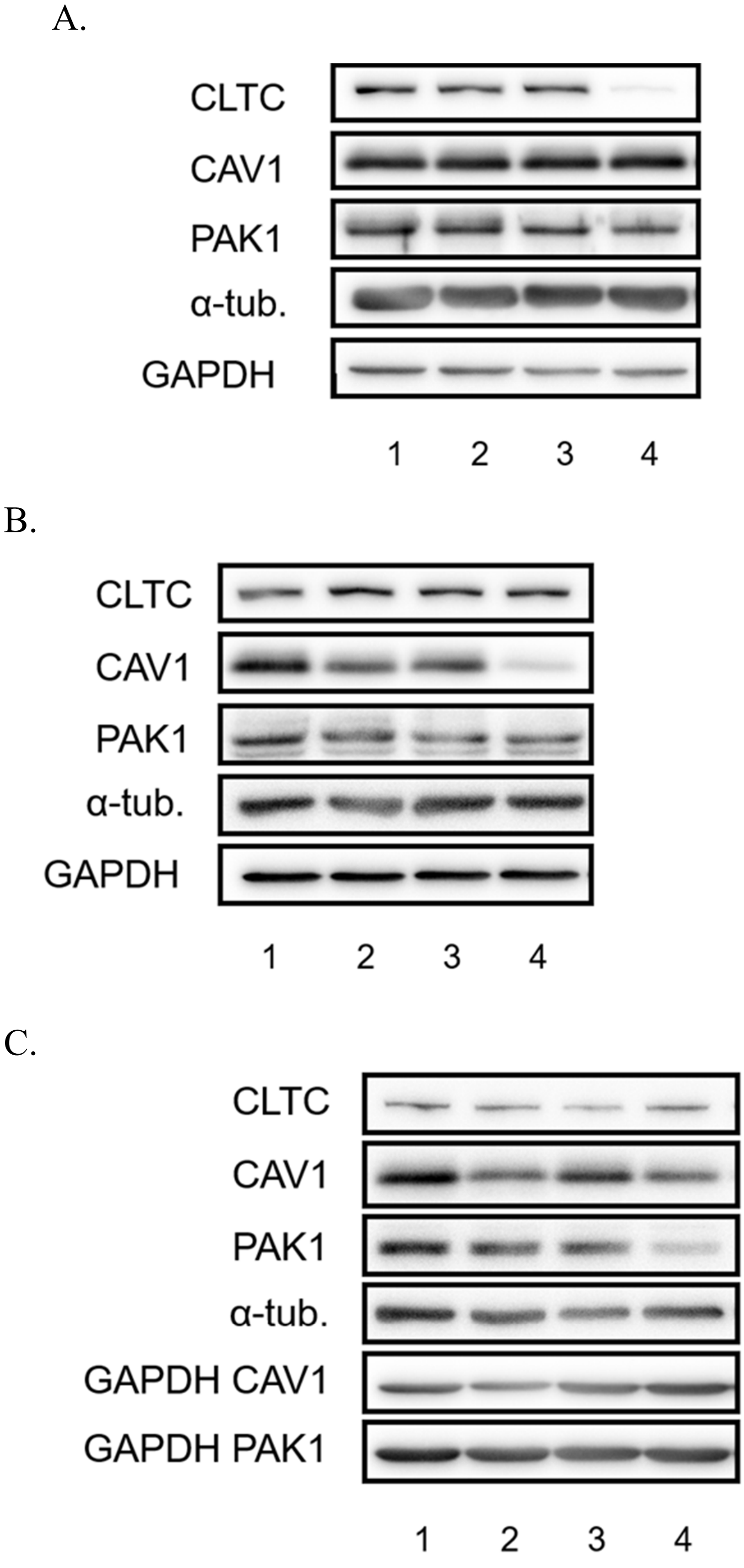

© 2020 by the authors. Licensee MDPI, Basel, Switzerland. This article is an open access article distributed under the terms and conditions of the Creative Commons Attribution (CC BY) license (http://creativecommons.org/licenses/by/4.0/).
